# The effect of femoral and popliteal percutaneous transluminal balloon angioplasty on patients’ quality of life

**DOI:** 10.1590/S1516-31802007000400012

**Published:** 2007-07-01

**Authors:** Ladislav Slovacek, Birgita Slovackova, Vendelin Chovanec

**Keywords:** Quality of life, Balloon angioplasty, Popliteal artery, Femoral artery, Atherosclerosis, Qualidade de vida, Angioplastia com balão, Artéria poplítea, Artéria femoral, Aterosclerose

## Abstract

**CONTEXT AND OBJECTIVE:**

Peripheral arterial occlusive disease (PAOD) is a prevalent atherosclerotic disorder characterized by limb pain on exertion, limb loss and a high mortality rate. Because of its chronic nature, it often has a negative impact on patients’ quality of life (QOL). This study aimed to assess QOL among patients with PAOD that was treated by endovascular intervention using femoral and popliteal percutaneous transluminal balloon angioplasty (PTBA).

**DESIGN AND SETTING:**

This study was local, prospective and longitudinal. It was carried at the Second Department of Internal Medicine of Charles University Hospital in Hradec Kralove, Czech Republic.

**METHODS:**

Thirty PAOD patients (20 male and 10 female) were treated by endovascular intervention using femoral and popliteal PTBA. The Czech version of the international generic European Quality of Life Questionnaire (EQ-5D) was applied.

**RESULTS:**

The statistical evaluation demonstrated that QOL presented highly significant statistical dependence on femoral and popliteal PTBA (p < 0.0001).

**CONCLUSION:**

The results showed that femoral and popliteal PTBA had a highly positive effect on the QOL of patients with PAOD.

## RESUMO

**CONTEXTO E OBJETIVO:**

Doença arterial oclusiva periférica (DAOP) é uma desordem aterosclerótica prevalente, caracterizada por dor esforço do membro, perda do membro e alta taxa de mortalidade. Devido à sua natureza crônica, freqüentemente tem impacto negativo na qualidade de vida (QV) dos pacientes. Este estudo objetivou avaliar a QV entre pacientes com DAOP tratada por intervenção endovascular usando angioplastia com balão transluminal femoral e poplítea (ABT).

**TIPO DE ESTUDO E LOCAL:**

Este estudo local, prospectivo e longitudinal foi realizado no Segundo Departamento de Medicina Interna do Charles University Hospital em Hradec Kralove, República Checa.

**MÉTODOS:**

30 pacientes com DAOP (20 homens) foram tratados por intervenção endovascular usando ABT femoral e poplítea. A versão checa do Questionário Europeu de Qualidade de Vida (EQ-5D) foi aplicada.

**RESULTADOS:**

A avaliação estatística demonstrou que a QV apresentou dependência altamente significativa da ABT femoral e poplítea (p < 0,0001).

**CONCLUSÃO:**

Os resultados mostraram que a ABT femoral e poplítea teve efeito altamente positivo na QV dos pacientes com DAOP.

## INTRODUCTION

Peripheral arterial occlusive disease (PAOD) is a prevalent atherosclerotic disorder characterized by limb pain on exertion, limb loss and a high mortality rate. In the Czech population, the prevalence of PAOD among men under 50 years old is less than 2%, and this level is reached 10 years later by Czech women. Intermittent claudication is the most common symptom in patients with PAOD.^1^ Endovascular revascularization (artery balloon and stent angioplasty) currently serves as an effective therapeutic method for patients with high-grade stenosis of the proximal limb artery segments.^1^ Surgical revascularization is usually reserved for patients who present severe aortoiliac disease and have low cardiovascular perioperative ischemic risk, and for whom long-term patency is likely to be achieved. All patients with PAOD of any severity need to achieve reductions in their atherosclerosis risk factors down to normal levels and use antiplatelet therapies.^1^ PAOD is a chronic disease that is associated with physical, psychological and social distress for elderly patients and their families.^2^ People with PAOD present significant disability that also affects the psychosocial and emotional aspects of their quality of life (QOL).^3^

QOL is defined as a patient's subjective evaluation of his or her life situation.^4^ QOL measurements contains information on an individual's physical, psychological, social and spiritual condition. QOL is evaluated by means of generic and specific questionnaires. Generic QOL questionnaires generally evaluate a patient's overall condition, regardless of the disease involved. Specific QOL questionnaires are designed to evaluate a patient's overall condition in relation to a particular type of disease. Modules are often used with these specific questionnaires. These modules are focused on specific symptoms and complaints relating to a particular type of disease.^4^

## OBJECTIVE

To evaluate the effect of femoral and popliteal percutaneous transluminal balloon angioplasty (PTBA) on QOL, among patients with PAOD.

## MATERIAL AND METHODS

We carried out this prospective longitudinal study among 30 patients with PAOD who were treated using femoral and popliteal PTBA during 2006 (January 1 to June 1), at the Second Department of Internal Medicine of Charles University Hospital in Hradec Kralove, Czech Republic. We evaluated the effect of this procedure on these patients’ QOL. For all of these PAOD patients, there was involvement of the femoral and popliteal arterial circulation. All of these 30 patients were aged over 18 years. This study was approved by Charles University Hospital Ethics Committee.

These patients’ QOL was assessed by means of the Czech version of the international generic European Quality of Life Questionnaire (EQ-5D).^4^ Statistical analysis was performed by means of analysis of variance (ANOVA) and the paired t-test. Descriptive analysis was used to evaluate the QOL questionnaire. p values < 0.05 were considered significant. The statistical analysis was conducted using the StatSoft Statistica Base software package, version 7.1.

## RESULTS

The total number of respondents with PAOD was 30 (20 males and 10 females). The respondents were classified according to the Fontaine system as follows: stage IIA (intermittent claudication; pain-free walking distance > 200 m), three patients; stage IIB (intermittent claudication; pain-free walking distance < 200 m), twelve patients; stage II (complicated) (skin lesions and noncritical limb ischemia; ankle pressure > 50 mmHg and/or toe pressure > 30 mmHg), six patients; stage III (chronic critical limb ischemia; pain while resting), four patients; and stage IV (chronic critical limb ischemia; ischemic lesions: ulcer, gangrene and necrosis), five patients. The mean age for all 30 respondents was 63.1 years (age range: 49 - 79 years). The mean time interval since femoral and popliteal PTBA was 3.8 months (range: 3 - 6 months).

The results showed that femoral and popliteal PTBA had a highly positive effect on QOL among these respondents with PAOD. The mean EQ-5D score (QOL dimensions) before femoral and popliteal PTBA for all 30 respondents with PAOD was 66.7%. The mean value for EQ-5D VAS (visual analog scale; subjective health status) before femoral and popliteal PTBA was 63.3%. The mean EQ-5D score (QOL dimensions) 3.8 months after femoral and popliteal PTBA was 72.3%. The mean value of EQ-5D VAS (subjective health status) 3.8 months after femoral and popliteal PTBA was 71%.

The statistical evaluation demonstrated that QOL (EQ-5D score and EQ-5D VAS) presented highly significant statistical dependence on femoral and popliteal PTBA (p < 0.0001) (Table 1).

**Table 1.** Comparison of mean European Quality of Life Questionnaire (EQ-5D) score values and mean EQ-5D visual analog scale (VAS) values before femoral and popliteal percutaneous transluminal balloon angioplasty (PTBA) and three to six months after femoral and popliteal PTBA (n = 30; p < 0.0001)

Quality of life	Before femoral and popliteal PTBA	3-6 months after femoral and popliteal PTBA
Mean EQ-5D score value (%)	66.7	72.3
Standard deviation	9.4	7.8
Mean EQ-5D VAS value (%)	63.3	71
Standard deviation	11.8	8.2

## DISCUSSION

Our results have shown the existence of an association between endovascular intervention by means of femoral and popliteal PTBA and overall QOL among patients with PAOD. Endovascular intervention by means of femoral and popliteal PTBA significantly improved QOL among these patients.

It is common in clinical medical practice to evaluate a patient's health condition and the success of the treatment on the basis of a single type of marker, most often by means of somatic, laboratory or detection markers. But the trend in modern clinical medicine is to evaluate a patient's health condition in a more complex way, using other characteristics. Use of QOL leads to an assessment that includes more dimensions of a number of aspects of life. Different aspects of life may be affected in different ways at different phases of the disease and its treatment. That is why this information enriches our knowledge of patients’ needs and it can significantly contribute towards the improvement of medical treatment. It can also help to reveal the mechanisms that modify the origin and course of the disease.^4^

Our study is the first investigation of QOL among patients with PAOD who were treated by means of PTBA in our country. Our study is one of the few such studies carried out in countries within the former Eastern European bloc.

We are, however, also aware of the fact that our study may present limitation due to a few other factors:

1. There were a relatively small number of patients in our group with PAOD. Therefore, we did not compare the patients’ QOL with regard to intermittent claudication versus chronic critical limb ischemia. Also, we did not compare the QOL among PAOD patients treated by means of PTBA with the QOL among PAOD patients treated conservatively.
2. There was a lack of patients with PAOD treated by means of stent PTBA.

## CONCLUSION

Our study demonstrates that femoral and popliteal PTBA present a positive effect on QOL among patients with PAOD.
